# The Release of Nitric Oxide from S-Nitrosothiols Promotes Angiogenesis

**DOI:** 10.1371/journal.pone.0000025

**Published:** 2006-12-20

**Authors:** Bahjat Al-Ani, Peter W. Hewett, Suborna Ahmed, Melissa Cudmore, Takeshi Fujisawa, Shakil Ahmad, Asif Ahmed

**Affiliations:** 1 Department of Reproductive and Vascular Biology, Centre for Cardiovascular Sciences, Institute of Biomedical Research, Medical School, University of Birmingham Birmingham, United Kingdom; 2 Birmingham Women's Hospital NHS Trust Birmingham, United Kingdom; Baylor College of Medicine, United States of America

## Abstract

**Background:**

Free nitric oxide (NO) reacts with sulphydryl residues to form S-nitrosothiols, which act as NO reservoirs. We sought to determine whether thiol-preserving agents and antioxidants, such as dithiothreitol (DTT) and vitamin C, induce NO release from S-nitrosylated proteins in endothelial cell cultures to promote angiogenesis.

**Methodology/Principal Findings:**

NO release was measured directly in cell supernatants using a Sievers NO Analyser, and *in vitro* angiogenesis was assessed by quantifying capillary-like tube network formation of porcine aortic endothelial cells (PAEC) on growth factor-reduced Matrigel. Incubation of PAEC with DTT or vitamin C significantly increased NO release in a concentration-dependent manner. However, the nitric oxide synthase (NOS) inhibitors, L-NNA and L-NIO, had no effect on DTT- or vitamin C-induced NO release, and there was no concomitant increase in the phosphorylation of endothelial NOS at serine-1177 following DTT or vitamin C treatment. DTT and vitamin C increased capillary-like tube network formation by nine- and two-fold, respectively, and the addition of copper ions doubled the effect of vitamin C. Surprisingly, DTT maintained endothelial tube networks for up to one month under serum-free conditions, and selective inhibitors of guanylyl cyclase (ODQ) and PKG (KT-5823) blocked this, demonstrating the requirement of cyclic GMP and PKG in this process.

**Conclusions/Significance:**

Both DTT and vitamin C are capable of releasing sufficient NO from S-nitrosothiols to induce capillary morphogenesis. This study provides the first evidence that increased denitrosylation leads to increased bioavailability of NO, independent of NOS activity, to promote sustained angiogenesis.

## Introduction

Nitric oxide (NO), generated by endothelial NO synthase (eNOS), is a key regulator of vascular function [1]. The physiological actions of NO are mediated predominantly via the activation of soluble guanylyl cyclase leading to generation of the potent second messenger cyclic guanosine monophosphate (cGMP) from guanosine 5′-triphosphate (GTP) [2], [3]. Cyclic GMP in turn activates cGMP-dependent kinases, such as protein kinase G (PKG), culminating in the regulation of many functions including the control of vascular tone, inhibition of platelet aggregation and neutrophil adhesion to endothelium, and reduction of vascular smooth muscle cell proliferation [4], [5]. NO is also a critical mediator of vascular endothelial cell growth factor (VEGF)-induced angiogenesis as VEGF fails to induce angiogenesis in eNOS^−/−^ knock-out mice [6], [7] and eNOS inhibitors block VEGF-induced angiogenesis [8], [9].

NO reacts rapidly with free sulphydryl groups to form S-nitrosothiols [10]. Circulating free NO has a very short half-life [11], [12], and the majority reacts with sulphydryl-containing proteins such as serum albumin that act as NO reservoirs dramatically increasing its half-life [10], [13]–[15]. Patients undergoing chronic hemodialysis have reduced NO activity due to increased levels of S-nitrosothiol-albumin, which represents an independent prognostic indicator of cardiovascular events [16]. S-nitrosoproteins have been detected in many cell types including endothelial cells [14], and S-nitrosylation is now recognised as a major post-translational modification that can affect the functional activity of proteins [17]. In addition, glutathione is the main non-protein that is S-nitrosylated in cells and extracellular fluids [18]. S-nitrosothiols are sensitive to reduction, and in the circulation they are denitrosylated by agents such as vitamin C, cysteine and reduced glutathione to release NO [19], [20]. Recent evidence from mice deficient in S-nitrosoglutathione reductase demonstrates that it is essential for S-nitrosothiol metabolism and highlights the critical role of S-nitrosothiols in NO biology and vascular homeostasis [21]. However, the significance of NO released from S-nitrosothiol reservoirs, in response to stimulation with thiol preserving agents and antioxidants, on endothelial cell function and angiogenesis has not been evaluated.

In this study we examined the contribution of NO-release from S-nitrosothiols on *in vitro* angiogenesis. NO production was assayed directly in porcine aortic endothelial cells (PAEC) treated with DTT or vitamin C in the presence and absence of the NOS inhibitors, L-NG Nitroarginine (L-NNA) and L-N^5^-(-1-Iminoethyl)ornithine dihydrochloride (L-NIO) using a Sievers NOA 280 chemiluminescence analyzer. Both DTT and vitamin C were found to produce sufficient NO-release, in the absence of NOS activity, to promote the formation of endothelial cell capillary-like tube networks.

## Methods

### Cell Culture

PAEC were grown in full growth medium consisting of HAM F12 nutrient mixture supplemented with 25 mm l-glutamine, 100 U/ml Penicillin and 100 µg/ml Streptomycin sulphate and 10% fetal calf serum (FCS) and cultured as described previously [9]. All cell culture reagents were obtained from Sigma (Poole, Dorset, UK) unless stated otherwise.

### Measurement of NO Release

PAEC were seeded at 1×10^5^ cells per well in 24-well plates containing full growth medium. All stimulations were performed with confluent quiescent cell monolayers in serum-free medium consisting of HAM F12 nutrient mixture supplemented with 25 mm l-glutamine, 100 U/ml penicillin and 100 µg/ml streptomycin sulphate and 0.2% bovine serum albumin (BSA). Cells were treated with various concentrations of DTT (Sigma) or vitamin C (Aldrich) in a final volume of 0.5 ml at 37°C for 60 minutes and the supernatants harvested and stored at −80°C for NO analysis. For the experiments using pharmacological inhibitors, PAEC were pre-incubated for 30 minutes with L-NNA or L-NIO (Merck Biosciences), at the concentrations indicated, prior to DTT or vitamin C stimulation. NO was measured directly in the gas phase, using a Sievers NOA 280 chemiluminescence analyzer (Analytix, Sunderland, UK) as described previously [22]. The level of NO detected in cell supernatants were corrected for background by subtracting the amount of NO present in serum-free endothelial cell culture medium incubated under the same conditions in the absence of PAEC.

### Tube formation assay

The formation of capillary-like tubular structures was studied on growth factor-reduced Matrigel (Becton Dickinson Labware) diluted 1∶1 in ice-cold serum-free medium, as described previously [9]. PAEC were seeded at a density of 6×10^4^ cells per well on Matrigel-coated 24 well dishes in full growth medium for 2 hours at 37°C. The growth medium was removed and DTT, vitamin C or the vehicle alone added in serum-free medium containing 0.2% bovine serum albumin (BSA). For the studies using pharmacological inhibitors, cells were pre-treated with test substances as indicated at 37°C for 30 minutes prior to stimulation with DTT or vitamin C. Capillary-like tube formation was observed with a Nikon inverted microscope and the results recorded digitally using Optimas image analysis software (Media Cybernetics).

### Western blotting

Confluent PAEC grown on 6-well plates were treated as indicated in medium containing 0.2% BSA for 15 minutes at 37°C. The cells were lysed on ice in RIPA buffer (UpState) containing phosphatase and protease inhibitors (Sigma) and insoluble protein removed by centrifugation at 13,000 g for 5 min. The protein concentration of samples was estimated using the DC Protein Assay (BioRad) and 30 µg protein from each lysate was mixed with 2× Llamelli sample buffer, separated by 10% SDS–PAGE and semi-dry electroblotted onto nitrocellulose membrane. The blots were blocked for 1 hour in 10% fat-free milk powder in Tris-buffered saline containing 0.1% Tween 20 (TBS-T) and the membranes incubated with a 1∶1000 dilution of rabbit anti-phospho-serine-1177 eNOS or anti-eNOS polyclonal antibodies, (Cell Signalling) overnight at 4°C. After washing with TBS-T, the membranes were incubated for 1 hour with a 1∶5000 dilution of peroxidase-labelled goat anti-rabbit polyclonal antibodies (Vector Laboratories) at room temperature. The membranes were then washed with TBS-T, and antibody binding visualised by enhanced chemiluminescence (Amersham Biosciences).

### Statistics

All data are expressed as the mean±SEM. Statistical analysis was performed using the two-tailed Student's t-test and p<0.05 was considered statistically significant.

## Results

### NO release in endothelial cells in the absence of eNOS activation

**Figure 1:**
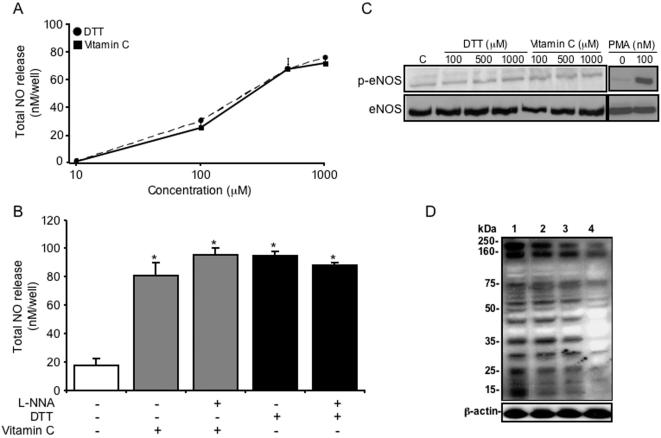
**The effect of DTT and vitamin C on NO-release and eNOS activation in endothelial cells**. (**A**): Porcine aortic endothelial cells (PAEC) were grown to confluence in 24-well plates and stimulated with increasing concentrations of DTT and vitamin C for 1 hour and NO measured in the supernatants. (**B**): Total NO release from PAEC following 1 hour incubation with 500 µM DTT and vitamin C in the absence or presence of the NOS inhibitor, L-NNA (500 µM). NO was measured directly using a Sievers NOA 280 chemiluminescene analyzer and the results were corrected for background levels of NO present in the medium. Results are the mean (±SEM, *bars*) of two experiments (n = 12), *p<0.01 *versus* control. (**C**): Western blots showing the effect of increasing concentrations of DTT, vitamin C or phorbol myristate acetate (PMA) on the phosphorylation of eNOS (phospho-eNOS) at serine-1177 in PAEC after 15 min. (**D**): Western blot showing the presence of S-nitrosylated proteins of PAEC pre-treated with 30 µM SNAP (lanes 1–3) or vehicle (lane 4) for 1 hour and then incubated with 500 µmol/L DTT (lane 2) or 500 µM vitamin C (lane 3) or vehicle (lanes 1 and 4) for 1 hour.

To investigate the effect of DTT or vitamin C on NO-release in endothelial cells, PAEC were grown to confluence and incubated with varying concentrations of DTT and vitamin C for 1 hour. Both DTT and vitamin C induced a similar concentration-dependent increase in NO release from PAEC cultures (Fig. 1A). Following treatment with 500 µmol/l DTT or vitamin C NO-release was found to be 400±5.55% and 344.5±12.5% respectively, above control values (Fig. 1B).

Endothelial NOS is the major source of NO produced by endothelial cells [1]. To determine whether the NO released following incubation of PAEC with DTT or vitamin C is generated by eNOS, we pre-incubated PAEC with the eNOS inhibitors L-NNA (500 µM) or L-NIO (10 µM) for 30 minutes prior to the addition of DTT or vitamin C. Interestingly, both L-NNA (Fig. 1B) and L-NIO (data not shown) failed to block the NO release induced by DTT or vitamin C, indicating that the increase in NO release was independent of eNOS activity. Consistent with these findings DTT and vitamin C did not stimulate a detectable increase in phosphorylation of eNOS at serine-1177, which is required for its activation (Fig. 1C) [23]. In contrast, PAEC stimulated with the PKC activator, phorbol myristate acetate (PMA; 100 nmol/l) showed a 5-fold increase in eNOS phosphorylation (Fig. 1C).

Finally, we examined the effect of DTT and vitamin C on the level of S-nitrosothiols in PAEC. Cells were incubated with 500 µM DTT or 500 µM vitamin C for 1 hour, and the extracted proteins Western blotted with an anti-S-nitrosothiol antibody. The blots show S-nitrosylated proteins ranging from approximately 15 to 250 kDa in PAEC (Fig. 1D). Pre-incubation of PAEC with the NO donor, SNAP, increased the level of S-nitrosoproteins (Fig. 1D) in these cells, which was reduced in some of these proteins by the addition of 500 µM DTT, or 500 µM vitamin C for 1 hour (Fig. 1D). The presence of biologically relevant transition metals such as copper has been shown to promote the decomposition of S-nitrosothiols mediated by vitamin C [24], [25]. Addition of 100 µM CuSO_4_ with vitamin C to PAEC produced a 20% increase in NO release over that of vitamin C alone (data not shown). This further supports our findings indicating that this NO release is occurring independently of eNOS activation.

### DTT and vitamin C induce capillary-like tube formation

**Figure 2:**
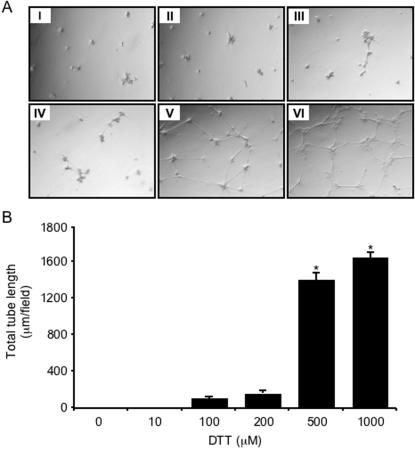
**The effect of DTT on** ***in vitro*** **tube formation**. **A** Endothelial cells grown on growth factor-reduced Matrigel were stimulated with (I) the vehicle alone (control) and with (II) 10, (III) 100, (IV) 200, (V) 500, and (VI) 1000 µM DTT. Cells were observed 24 hours after stimulation and results recorded digitally. **B** Quantitative analysis of the results representing the mean total tube length per 40× field. The results are the mean (±SEM, *bars*) of three different fields. *, *p*<0.01 *versus* 200 µM DTT.

To investigate whether the observed increase in free NO following incubation with DTT or vitamin C can affect endothelial cell function, we plated PAEC on growth factor reduced Matrigel and examined their ability to form capillary-like tube networks. The degree of network formation was quantified by measuring the mean total tube length per 40× field. Increasing the concentration of DTT above 10 µM produced a concomitant increase in the degree of arrangement of PAEC into tubular networks (Fig. 2). At 1 mM DTT, PAEC reorganised into complete tubular networks (Fig. 2A VI), whereas no tube formation was observed with 10 µmol/l DTT (Fig. 2A II) or under control conditions (Fig. 2A I). There was a ∼15-fold increase in mean total tube length (Fig. 2B, p<0.01, n = 5) with 1 mM DTT compared with that observed with 200 µM DTT.

**Figure 3:**
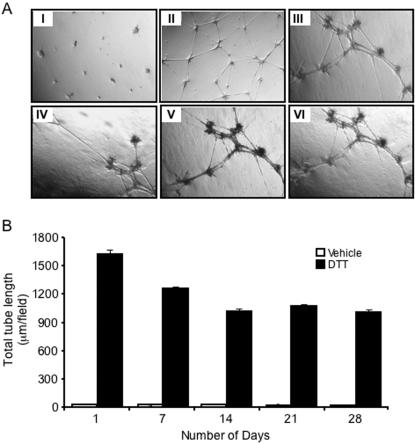
**The long-term effect of DTT on capillary-like tube formation**. **A** Porcine aortic endothelial cells were seeded on growth factor-reduced Matrigel and stimulated with the vehicle alone for 1 day (I) or 1 mM DTT (II–V) and observed at (II) 1, (III) 7, (IV) 14, (V) 21, and (VI) 28 days. **B** Histograms showing the quantitative analysis of the mean total tube length per 40× field. Results are the mean (±SEM, *bars*) of three different fields. *, *p*<0.01;**, *p*<0.001 *versus* control.

As DTT strongly induced the organisation of PAEC into tube networks, we investigated the long-term effect of 1 mM on *in vitro* angiogenesis. Interestingly, DTT maintained PAEC tube networks for approximately one month in serum-free medium. Over time we observed a significant increase in the thickness of the tube structures (Fig. 3A II–VI) and concomitant reduction in mean total tube length (Fig. 3B; P<0.05, n = 5).

**Figure 4:**
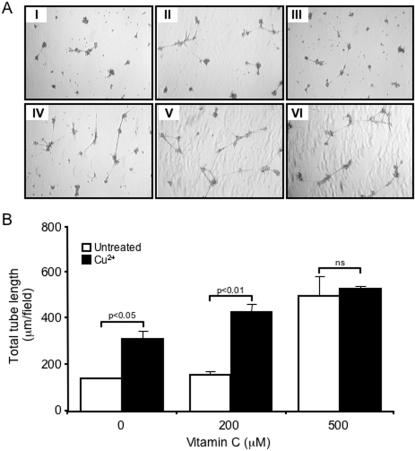
**The effect of vitamin C on capillary-like tube formation**. **A** Porcine aortic endothelial cells seeded on growth factor-reduced Matrigel were stimulated with either the vehicle alone (I), 10 µM CuSO_4_ (II), 200 µM vitamin C (III), 200 µM vitamin C and 10 µM CuSO_4_ (IV), 500 µmol/L mmol/L vitamin C (V) and 500 µM vitamin C and 10 µM CuSO_4_ (VI), for 24 hours. **B** Histogram showing the mean total tube length per 40× field. The results are the mean (±SEM, *bars*) of three different fields of three independent experiments.

The effect of increasing concentrations of vitamin C on the formation of tubular structures by PAEC was monitored in the presence of 10 µM copper. After 24 hours incubation, vitamin C at 500 µM stimulated a 2-fold increase in tube length (Fig. 4). The capillary networks collapsed and the cells dispersed into the surrounding medium after 48 hours (data not shown). The addition of 10 µM copper sulphate alone induced a 2-fold increase in total tube length compared with untreated cells, which was augmented in the presence of 200 µmol/l vitamin C (Fig. 4B). In contrast to DTT, vitamin C produced incomplete tube networks consisting of short tubular-like structures, even at concentrations of 1 mM (Fig. 4A and data not shown).

### DTT- induced tube formation is dependent on guanylyl cyclase and PKG activity

**Figure 5:**
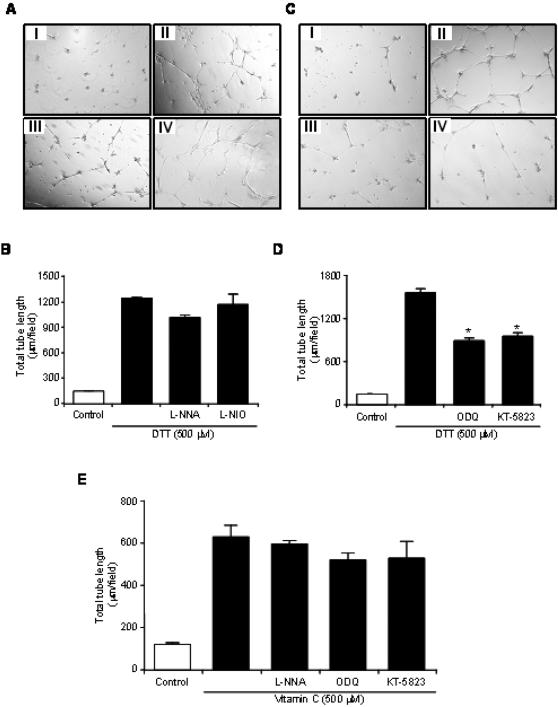
**DTT-induced capillary-like tube formation is dependent on guanylyl cyclase and PKG activity**. Porcine aortic endothelial cells (PAEC) plated on growth factor-reduced Matrigel were pre-incubated for 30 minutes with the NOS inhibitors, L-NNA (500 µM) and L-NIO (10 µM), guanylyl cyclase inhibitor, ODQ (10 µM), or PKG inhibitor, KT-5823 (1 µM), at 37°C in medium containing 0.2% BSA prior to stimulation with 500 μM DTT or vitamin C for 24 hours. **A** Vehicle alone (I), DTT (II), DTT and L-NNA (III), DTT and L-NIO (IV), **B** Histogram showing the mean total tube length per 40× field of PAEC following stimulation with DTT in the presence of L-NNA and L-NIO following 24 hour incubation. **C** Vehicle alone (I), DTT (II), DTT and ODQ (III), DTT and KT-5823 (IV), **D** Histogram showing the mean total tube length per 40× field of PAEC following stimulation with DTT in the presence of ODQ or KT-5823 following 24 hour incubation. **E** Histogram showing the mean total tube length per 40× field of PAEC following incubation with vitamin C in the presence of L-NNA, L-NIO, ODQ or KT-5823. The results are the mean (±SEM *bars*) of three different fields.

To investigate the mechanism(s), by which DTT and vitamin C promote capillary-like tube formation, PAEC were pre-incubated for 30 minutes with inhibitors of eNOS (L-NNA and L-NIO), guanylyl cyclase (ODQ) and PKG (KT-5823) prior to stimulation. The results of these experiments are summarised in Fig. 5 and show that the tube formation stimulated by DTT (Fig. 5A and B) or vitamin C (Fig. 5E) is independent of eNOS activity. As many of the activities of NO are dependent on guanylyl cyclase and its downstream effector PKG we investigated their effect on DTT and vitamin C induced tube formation. ODQ (10 µM) and KT-5823 (1 µM) significantly reduced the ability of DTT to induce tube formation, implicating the guanylyl cyclase-PKG pathway in this process (Fig. 5C and D). In contrast to DTT, the effect of vitamin C was not abrogated by the same concentration of ODQ and KT-5823 indicating that guanylyl cyclase and PKG are not required for its activity (Fig. 5E).

## Discussion

In this study we show that the antioxidants DTT and vitamin C can release sufficient NO from PAEC cultures to promote *in vitro* angiogenesis via cGMP dependent and independent pathways. The pro-angiogenic activity of several growth factors, including the VEGF family and angiopoietins, is dependent on eNOS activity [6]–[9], [26]. The requirement of NO for VEGF-mediated angiogenesis was highlighted by studies of eNOS^(−/−)^ knock-out mice in which VEGF fails to induce neovascularisation [6], [7]. Similarly, VEGF-stimulated endothelial cell capillary-like tube network formation *in vitro* requires activation eNOS leading to NO release and is reported to be dependent on the subsequent generation of the second messenger cGMP [8], [9]. Consistent with these findings the rearrangement of PAEC into capillary-like tube networks in response to DTT was found to be dependent on guanylyl cyclase and PKG activity confirming that cGMP is a key mediator of *in vitro* angiogenesis.

Thiol depletion has been shown to inhibit eNOS activity and subsequent NO production in endothelial cells [27] and the rat aorta [28]. In human umbilical vein endothelial cells the removal of glutathione with 1-chloro-2,4-dinitrobenzene dramatically decreased the generation of citrulline, a by-product of NO synthesis, and was reversed by the addition of reduced glutathione [27]. Similarly, the oxidation of intracellular thiols in PAEC using diamide was reported to result in a 75% inhibition of eNOS activity, which was completely restored by DTT [29]. In addition, DTT was shown to directly activate oxidised purified bovine eNOS *in vitro* [29]. However, in our assays PAEC were not deliberately exposed to thiol depleting agents or direct oxidative stress. In addition, DTT- and vitamin C-mediated NO-release was not blocked by NOS inhibitors (Fig. 1) suggesting that NO was being released from S-nitrosothiol reservoirs.

S-nitrosoproteins have been detected in many cell types including the endothelium [14]. The presence of increasing concentrations of reducing agents such as vitamin C and cysteine leads to the rapid release of NO and progressive decrease in detectable S-nitrosoproteins [14], [19], [25]. This process is enhanced by the presence of biologically relevant reduced transition metals such as copper and iron [24], [25]. These findings are in general agreement with our observations of the release of NO from S-nitrosothiols in PAEC cultures following treatment with DTT and vitamin C (Fig. 1A and B) was independent of NOS activation. This was demonstrated by the increase of NO-release from endothelial cell cultures pre-treated with the NOS inhibitors L-NNA and L-NIO, and also a lack of eNOS phosphorylation at serine-1177, which is required for full activation of the enzyme [23], following stimulation with DTT and vitamin C. Furthermore, vitamin C-mediated NO-release and tube formation was enhanced by the addition of copper ions. This is consistent with the reported release of NO from S-nitrosoalbumin following the addition of vitamin C and free copper [25].

Relative differences in reducing activity may account for the profound difference in the ability of DTT, and vitamin C to induce and maintain tube formation. Vitamin C stimulated the formation of PAEC into tube networks, which were established following overnight incubation. The extent of vitamin C-induced tube network formation decreased after 24 hours and had disappeared by 48 hours. In contrast, DTT stimulated the formation of complete tubular networks that were strikingly maintained for up to a month. While DTT required guanylyl cyclase and PKG activity to induce tube formation (Fig. 5) the activity of vitamin C was independent of cGMP. The mechanisms underlying the long-term maintenance of DTT-stimulated tube networks require further investigation.

Our demonstration that vitamin C induces capillary-like tube formation are consistent with a previous study by May and colleagues [30] demonstrating that vitamin C enhances the synthesis of collagen type-IV in endothelial cells, which is associated with the initial phases angiogenesis involving endothelial cell migration and proliferation [31]. Conversely, Ashino and co-workers [32] report that vitamin C inhibited the formation of bovine pulmonary endothelial cell tube networks on growth factor-reduced Matrigel, at concentrations above 100 nM, and in the chorioallantoic membrane assay [33]. A possible explanation for this observation is that the endothelial cells were pre-treated with vitamin C for 30 minutes prior to plating onto Matrigel which may have limited their exposure to released NO, whereas in our study vitamin C was added to the cells following plating onto Matrigel and present for the duration of the experiment.

Antioxidants protect the body against the damaging effects of free radicals that can overwhelm the endogenous antioxidant systems in pathologies such as atherosclerosis and hypertension, and promote endothelial cell apoptosis [16], [33]–[36]. The oxidative depletion of vascular thiols increases the production of reactive oxygen species leading to apoptosis, which can be reversed by the addition of reducing agents such as DTT [37]–[39]. Reactive oxygen species can attenuate endothelium-dependent vasorelaxation [40], [41] and NO generated through the activation of eNOS can act as cardioprotective anti-oxidant preventing free radical damage [42]–[44]. Vitamin C is reported to inhibit the production of superoxide free radicals in pig coronary and rat mesenteric arteries [45], [46], promote blood vessel relaxation, lower blood pressure and preserve NO bioavailability in endothelial cells derived from apoE-deficient mice [47]. The clinical use of anti-oxidants in cardiovascular disease and cancer however remains controversial. Although small-scale clinical trails of vitamin C showed a significant improvement in endothelial function in patients with coronary artery disease [48] and congestive heart failure [49], large-scale randomised clinical trials of antioxidants in the prevention of cardiovascular disease and cancer have generated conflicting results. Vitamin C was reported to protect against stroke and coronary heart disease [50], [51], reduce blood pressure [52] and decrease mortality [53]. The most recent and comprehensive studies have shown no benefit from antioxidant therapy [54]–[56]. Our study indicates that antioxidants may promote angiogenesis through the release of NO from S-nitrosothiols which could lead to potentially adverse effects in pathologies such as cardiovascular disease where increased angiogenesis in the adventitia/neointima leads to plaque instability [57].
